# Learning by observation and learning by doing in Prader-Willi syndrome

**DOI:** 10.1186/s11689-015-9102-0

**Published:** 2015-02-26

**Authors:** Francesca Foti, Deny Menghini, Enzo Orlandi, Cristina Rufini, Antonino Crinò, Sabrina Spera, Stefano Vicari, Laura Petrosini, Laura Mandolesi

**Affiliations:** Department of Psychology, “Sapienza” University of Rome, Via dei Marsi 78, 00185 Rome, Italy; IRCCS Fondazione Santa Lucia, Via del Fosso di Fiorano 64, 00143 Rome, Italy; Child Neuropsychiatry Unit, Neuroscience Department, “Children’s Hospital Bambino Gesù”, Piazza Sant’Onofrio 4, 00100 Rome, Italy; Pediatric and Autoimmune Endocrine Disease Unit, “Children’s Hospital Bambino Gesù”, Palidoro, Via Torre di Palidoro, 00050 Fiumicino, Rome, Italy; Department of Motor Science and Wellness, University of Naples “Parthenope”, Via Medina 40, 80133 Naples, Italy

**Keywords:** Observational learning, Learning by trial and error, Imitation, Sequential learning, Genetic disorders, Social learning

## Abstract

**Background:**

New competencies may be learned through active experience (learning by doing) or observation of others’ experience (learning by observation). Observing another person performing a complex action accelerates the observer’s acquisition of the same action, limiting the time-consuming process of learning by doing. Here, we compared learning by observation and learning by doing in individuals with Prader-Willi syndrome (PWS). It is hypothesized that PWS individuals could show more difficulties with learning by observation than learning by doing because of their specific difficulty in interpreting and using social information.

**Methods:**

The performance of 24 PWS individuals was compared with that of 28 mental age (MA)- and gender-matched typically developing (TD) children in tasks of learning a visuo-motor sequence by observation or by doing. To determine whether the performance pattern exhibited by PWS participants was specific to this population or whether it was a nonspecific intellectual disability effect, we compared the PWS performances with those of a third MA- and gender-matched group of individuals with Williams syndrome (WS).

**Results:**

PWS individuals were severely impaired in detecting a sequence by observation, were able to detect a sequence by doing, and became as efficient as TD children in reproducing an observed sequence after a task of learning by doing. The learning pattern of PWS children was reversed compared with that of WS individuals.

**Conclusions:**

The observational learning deficit in PWS individuals may be rooted, at least partially, in their incapacity to understand and/or use social information.

**Electronic supplementary material:**

The online version of this article (doi:10.1186/s11689-015-9102-0) contains supplementary material, which is available to authorized users.

## Background

Prader-Willi syndrome (PWS) is a genetic disorder with an incidence rate at birth of about 1:15,000 to 1:20,000 caused by paternal deletion within 15q11-q13 (70% to 75% of cases), maternal disomy of chromosome 15 (mUPD) (20% to 25%), or unbalanced translocation or imprinting center defect (2%) [1,2]. PWS is characterized by hyperphagia; early-onset and morbid obesity if appropriate treatments (growth hormone treatment, diet and exercise regimes) are not provided; hypogonadism; hypotonia; maladaptive behavior, such as repetitive and stereotypical behavior, mental rigidity, impulsiveness, temper outbursts, and resistance to change; and impaired social functioning [3-5]. PWS individuals are characterized by a downward shift in the distribution of IQ scores and mild to moderate intellectual disability (ID) [6]. Their cognitive profile is characterized by strengths in long-term memory, visual perception, simultaneous processing, reading skill, and visuo-spatial functions and weaknesses in attention, short-term memory, sequential processing, executive functions, action-based visual processing, auditory processing, mathematical skills, language abilities, and social cognition [7-16].

Although the behavioral phenotype of PWS individuals has been characterized with regard to maladaptive behavior and cognitive profile (for a review, see [3]), their social functioning has been only recently examined. Social impairment exhibited by PWS individuals represents a deficit that is not merely a consequence of their maladaptive behavior, but it may reflect their specific difficulty in interpreting and using social information, such as emotional and nonverbal cues, facial emotional expressions, other’s mental and feeling states, and visual information into a coherent social story [16-19]. Most reports describe PWS people as characterized by poor peer relationships, social withdrawal, and preference for solitary activities [20,21]. Furthermore, they often display aggressive behavior and a deficitary comprehension of other’s thoughts or perspective [17,22].

To date, no research has analyzed whether different learning modalities facilitate or hinder the acquisition of new skills in PWS individuals. New competencies may be learned through active experience (learning by doing) or through observation of others’ experience (learning by observation) [23,24]. While learning by doing involves direct experience, learning by observation involves social processing, with all the other variables (for example, motor and cognitive complexity) being equal. Observing another person performing a complex action and solving a problem accelerates the observer’s acquisition of the same action, limits the time-consuming process of learning by trial and error, and reduces the practice needed to learn the skill [24,25]. Thus, it represents a powerful learning mechanism that may be based also on social processing [26-28].

The present research compared learning by observation and learning by doing in PWS individuals. It is hypothesized that PWS individuals show more difficulties with learning by observation than learning by doing because of their specific difficulty in interpreting and using social information.

**Figure 1:**
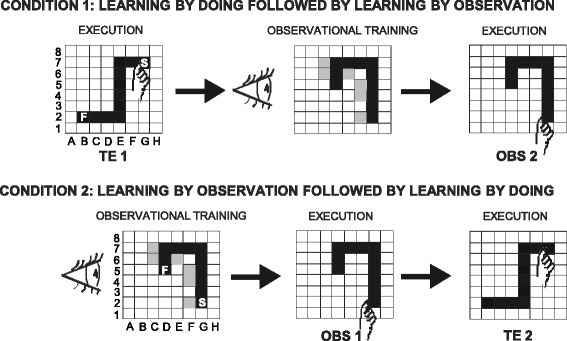
**Schematic diagrams of experimental conditions.** Condition 1: participants detected a sequence by doing (trial and error task, TE1), and after observational training, they reproduced the observed sequence (observational task, OBS2). Condition 2: after the observational training, participants reproduced the observed sequence (OBS1) and detected by doing a different sequence (TE2). The incorrect positions touched by the actor during the observational training are shown in gray. F: final point; S: starting point.

The participants learned a visuo-motor sequence by performing the task after observing an actor detect the sequence of correct items by trial and error (learning by observation) or by actually detecting the correct sequence by trial and error (learning by doing) (Figure 1). The same visuo-motor task was previously used in studies of individuals with Williams syndrome (WS), dyslexia, and autistic spectrum disorders [29-31]. The task is suitable for studying the declarative and procedural components of learning. The performances of PWS individuals were compared with those of a mental age (MA)- and gender-matched group of typically developing (TD) children. To determine whether the performance pattern exhibited by PWS participants was specific to this population or whether it was a nonspecific ID effect, we compared the PWS performances with those of a third MA- and gender-matched group of WS individuals [30]. The study design that matched three experimental samples allowed us to take into account whether the learning performance of PWS individuals was better or worse than expected given their general level of intellectual functioning indexed as MA (MA-matched control group) and whether the performances were due to the cognitive profile of their specific pathology considering IQ (ID-matched control group) [10,32].

## Methods

### Participants

Twenty-four individuals with PWS, 24 individuals with WS (syndromic control group), and 28 TD children (control group) matching the PWS and WS groups for MA and gender were examined. Eight individuals with WS and five TD children participated in both the present study as well as our recent research [30]. The stability of data displayed by WS participants was verified as described in Additional file 1: Table S1. The present study encompassed two experimental conditions: learning by doing followed by learning by observation (Condition 1) and learning by observation followed by learning by doing (Condition 2) (Figure 1; Table 1). The participants were randomly assigned to the two experimental conditions. Chronological age (CA) and MA, as well as IQ, of all participants are compared in Table 1.

**Table 1:** **Statistical comparisons (one-way ANOVA) of CA, MA, and IQ between PWS, WS, and TD groups**

**Group**	**CA Mean (±SEM)**	***F***_**(fd)**_ ***P*** ***η***_***P***_^**2**^	**MA Mean (±SEM)**	***F***_**(fd)**_ ***P*** ***η***_***P***_^**2**^	**IQ Mean (±SEM)**	***F***_**(fd)**_ ***P*** ***η***_***P***_^**2**^
PWS1 (Condition 1)	21.08 (±2.06)	*F*_1, 22_ = 1.16, *P* = 0.29, *η*_*P*_^2^ = 0.05	6.02 (±0.01)	*F*_1, 22_ = 0.70, *P* = 0.41, *η*_*P*_^2^ = 0.03	51.8 (±2.6)	*F*_1, 22_ = 0.07, *P* = 0.79, *η*_*P*_^2^ = 0.003
PWS2 (Condition 2)	18.05 (±1.08)		6.05 (±0.03)		52.7 (±2.9)	
WS1 (Condition 1)	20.05 (±2.04)	*F*_1, 22_ = 0.59, *P* = 0.45, *η*_*P*_^2^ = 0.03	6.04 (±0.02)	*F*_1, 22_ = 0.07, *P* = 0.78, *η*_*P*_^2^ = 0.003	54.2 (±2.7)	*F*_1, 22_ = 0.01, *P* = 0.90, *η*_*P*_^2^ = 0.0006
WS2 (Condition 2)	17.06 (±2.02)		6.05 (±0.03)		53.8 (±2.2)	
TD1 (Condition 1)	6.06 (±0.02)	*F*_1, 26_ = 0.03, *P* = 0.85, *η*_*P*_^2^ = 0.001	6.06 (±0.03)	*F*_1, 26_ = 0.69, *P* = 0.41, *η*_*P*_^2^ = 0.03	103 (±3.1)	*F*_1, 26_ = 2.27, *P* = 0.14, *η*_*P*_^*2*^ = 0.08
TD2 (Condition 2)	6.07 (±0.02)		6.04 (±0.03)		109.1 (±2.7)	

All pathological participants were part of a larger pool of individuals attending the Children’s Hospital Bambino Gesù for clinical and rehabilitative follow-up. In the PWS and WS participants, clinical diagnosis was confirmed by genetic investigation (fluorescence *in situ* hybridization (FISH)), which showed paternal deletion on chromosome band 15q11-q13 in the PWS group and deletion on chromosome band 7q11.23 in the WS group. All PWS participants had been receiving growth hormone therapy for ≥3 years and were in euthyroidism. All participants lived with their own families.

While TD children were individually tested in a quiet room at their schools, all syndromic participants were tested in a quiet room at the Children’s Hospital Bambino Gesù. The study was conducted according to the Declaration of Helsinki. The parents of participants gave written informed consent.

### Intelligence evaluation and neuropsychological assessment

The brief version of the Leiter International Performance Scale–Revised (four out of 10 subtests: figure ground, form completion, sequential order, and repeated patterns) was used to compute brief IQ and the corresponding MA [33]. Visuo-motor integration and memory functions were assessed by visuo-motor integration (VMI) [34], visuo-spatial short-term memory (VSS), and visuo-object short-term memory (VOS) tests [35]. Description of tests and statistical comparisons among groups are reported in Table 2 and Additional file 2: Table S2.

**Table 2:** **Statistical comparisons (one-way ANOVA) of performances of PWS, WS, and TD participants**

**Cognitive domain**	**PWS Mean (±SEM)**	**WS Mean (±SEM)**	**TD Mean (±SEM)**	**Group effect** ***F***_**(fd)**_ ***P*** ***η***_***P***_^**2**^	***Post hoc*** **Newman-Keuls’s test**
					***P*** **; Cohen’s** ***d*** **;** ***r***
VMI	13.08 (±0.54)	12.79 (±0.52)	15.14 (±0.28)	*F*_2, 73_ = 8.51	PWS vs. WS
				*P* = 0.0005	*P* = 0.65; *d* = 0.11; *r* = 0.05
				*η*_*P*_^2^ = 0.19	PWS vs. TD
					*P* = 0.002; *d* = −0.96; *r* = −0.43
					WS vs. TD
					*P* = 0.001; *d* = −1.12; *r* = −0.49
VSS	3.35 (±0.14)	2.63 (±0.19)	3.43 (±0.16)	*F*_2, 73_ = 6.50	PWS vs. WS
				*P* = 0.003	*P* = 0.004; *d* = 0.84; *r* = 0.39
				*η*_*P*_^2^ = 0.15	PWS vs. TD
					*P* = 0.75; *d* = −0.09; *r* = −0.05
					WS vs. TD
					*P* = 0.004; *d* = −0.87; *r* = −0.40
VOS	2.79 (±0.15)	2.71 (±0.13)	2.89 (±0.13)	*F*_2, 73_ = 0.48	
				*P* = 0.62	
				*η*_*P*_^2^ = 0.013	

VMI, visuo-motor integration; VSS, visuo-spatial short-term memory; VOS, visuo-object short-term memory.

### Experimental procedure

Each participant sat in front of a computer touch screen (distance 60 cm). In both conditions, the experimenter acting as the actor (FF) sat near the participant. An 8 × 8 black matrix appeared on the touch screen. The participant was asked to find a hidden sequence of correct squares prepared in advance by the experimenters. The sequence was composed of ten adjacent spatial positions in the matrix, which formed a snake-like pattern (Figure 1).

To explain the task to each participant, the experimenter used the same Italian verbal instructions because all participants were native Italian speakers. Below is the translation of the verbal instructions provided to all participants: ‘You have to find a snake formed by ten squares. When you touch a correct square belonging to snake body it will be turned gray and you will hear a sound; conversely, if you touch a wrong square not belonging to the snake, it will be turned red. In this case, you have to find a new gray square. You have to re-start each time you find a new correct square. After finding the whole snake, you have to re-touch it three times without making lighted red squares.’ The participants started touching a gray square, which was the first element of the sequence representing the snake body and was always lit up. In the search for the second correct square, the participants had to touch one of the four squares bordering the gray square by moving in the matrix vertically or horizontally, but never diagonally. Each touched square (correct or wrong) was lit up for 500 ms and then lighted off again; thus, no trace of the touched sequence remained on the screen.

In learning the sequence by trial and error (learning by doing), the participants tried to find the correct sequence immediately after the verbal instructions. Conversely, in learning the sequence by observation, after the verbal instructions the participants observed the actor while she (FF) detected a ten-item sequence by trial and error (observational training). The actor performed the task by always making the same errors in the same positions, so that all participants observed the same pattern of correct and wrong touches. No more than 2 min after the end of the observational training, the participants were required to reproduce the correct sequence (the snake).

### Parameters

Regardless of whether learning took place by observation or by doing, the two tasks involved three phases: the detection phase (DP) that ended once the participants found the tenth correct position, the exercise phase (EP) in which they had to repeat the ten-item sequence until their performance was error-free, and the automatization phase (AP) that ended when the correct sequence was repeated three consecutive times without errors.

The parameters measured were as follows: DP errors, calculated as the number of incorrect items touched in detecting the ten correct positions; EP repetitions, calculated as the number of replications needed to reach the error-free performance; and AP times (in ms), calculated as the time spent carrying out each of the three repetitions of the sequence. Considering DP and EP together, we calculated perseverations, consecutive errors touching the same square or a fixed sequence of squares; sequence errors, touching a correct square at the wrong moment (for example, touching E7 before F7); side-by-side errors, touching the squares bordering the correct sequence (for example, E8); illogical errors, touching any other square (for example, B5); and, exclusively in the observational learning task, imitative errors, touching the squares deliberately wrongly touched by the actor during the observational training (for example, F4) (Figure 1).

The error analysis allowed a multi-faceted characterization of the performance. Specifically, sequence and side-by-side errors allowed analysis of mnesic, planning, and inhibitory abilities, and cognitive flexibility. Illogical errors permitted analysis of adherence to the experimental setting and understanding the task instructions. Finally, imitative errors provided information on the tendency to adhere to the behavior of the social model (actor) and hyperimitate it, because the observational learning did not merely involve copying an action but required that the observer transformed the observation into an action as similar as possible to the model in terms of the goal (detecting the snake) to be reached. The hyperimitative tendency is faithfully copying both necessary and unnecessary actions made by the actor. Besides a reduced understanding of the rules of the task, hyperimitation may reflect a social process linked to the individual’s motivation to affiliate with the demonstrator or to closely conform to perceived norms [36,37]. Therefore, the analysis of the imitative errors is important to facet the features of the learning by observation.

### Condition 1: learning by doing followed by learning by observation

Twelve PWS, 12 WS, and 14 TD participants (Table 1) detected a sequence by doing (trial and error task, TE1), and after 10 min from task end, they observed the experimenter detect a different sequence (observational training). After 2 min, participants were required to reproduce the observed sequence (observational task, OBS2). There was no fixed time limit for executing the task.

Although the two sequences to be used as TE and OBS sequences had two different forms, their degree of difficulty did not differ because both sequences had the same number of squares (10) and corners (2). To confirm this assumption, a pilot study was conducted. Six TD children [four males] of MA 6.04 ± 0.2 years detected the two different sequences by doing; the presentation order was randomized among participants. DP errors made in detecting TE ($\overline{x}$_=_ 24.83 ± 2.57) and OBS ($\overline{x}$_=_ 20.83 ± 2.19) sequences, evaluated using Wilcoxon’s test, were not significantly different (*Z* = 1.21, *P* = 0.22).

### Condition 2: learning by observation followed by learning by doing

Twelve PWS, 12 WS, and 14 TD participants (Table 1) observed the experimenter detect a sequence (OBS1) and then reproduced it. After 10 min from task end, they detected a different sequence by doing (TE2). The difference between the two conditions was that participants reproduced a sequence learned by observation after (Condition 1) or before (Condition 2) the detection of a different sequence by doing. This protocol encompassing the use of both tasks (OBS and TE) in each condition allowed analysis of the performances of the same participants in the two types of learning. To exclude any practice effect, inevitably present in the second tasks and potentially affecting performances, Conditions 1 and 2 (with the only change being the order of presentation) were needed.

No significant differences in CA, MA, and IQ (always *P* > 0.1) among participants performing Conditions 1 and 2 were found (Table 1).

### Cognitive mapping abilities

In all participants, we evaluated the cognitive map, which was the spatial mental representation in which information about the relative locations of the squares was coded to connect them in the global sequence [38,39]. To this aim, at the end of each task (OBS or TE), every participant drew the arrangement of the just-reproduced sequence on an 8 × 8 matrix sketched on a paper sheet, in which only the starting point was indicated (Additional file 3). Each participant drew two sequences, one learned by observation and the other one by doing. We evaluated the positions of every square and considered error any marked square outside of the just-reproduced sequence. Three categories of errors were considered: no error, one error, and more than one error.

### Statistical analyses

The data were first tested for normality (Shapiro-Wilk’s test) and homoscedasticity (Levene’s test) and then compared by using two-, three-, or four-way analyses of variance (ANOVAs) followed by *post hoc* multiple comparisons by using Newman-Keuls’s test. The two-way ANOVAs were performed by applying the mixed model for independent variables (PWS, WS, and TD groups) and repeated measures (type of error: illogical, sequence, side-by-side, and imitative). Three-way ANOVAs (group × condition × task; group (PWS, WS, TD); condition (1, 2); task (OBS, TE)) were performed on DP errors, EP repetitions, and perseverations. A four-way ANOVA was performed on AP times by applying the mixed model for independent variables (group (PWS, WS, TD); condition (1, 2); task (OBS, TE)) and repeated measures (times (1, 2, 3) spent carrying out each of the three repetitions of the sequence). Error categories of mapping abilities were analyzed by the *χ*^2^ test. Data of the pilot study were analyzed by using nonparametric analysis (Wilcoxon’s test). Analyses were performed by Statistica 8.0, and the significance level was established at *P <* 0.05. Since in the present study a number of analyses was run, controlling for the alpha inflation was needed. We controlled the proportion of type I errors among all rejected null hypotheses by setting the false discovery rate (FDR) to 0.05. The FDR was estimated through the procedure described in [40]. In our results, the 0.05 level of significance corresponded to an FDR < 0.05. The complete statistical analyses are reported as Additional file 4: Table S4 and Additional file 5: Table S5.

## Results

### Learning tasks

**Figure 2:**
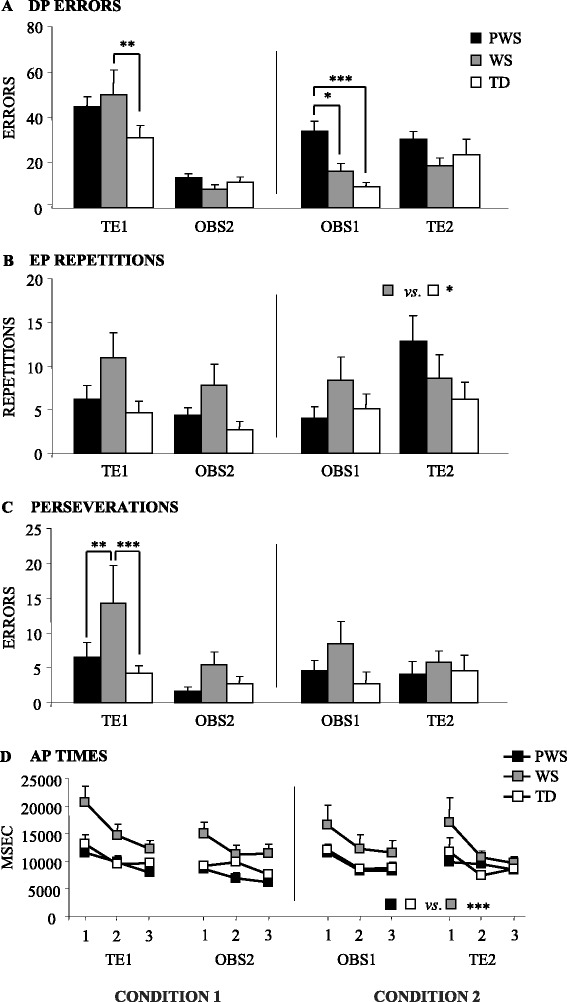
**Performances of PWS, WS, and TD participants. (A)** DP errors. **(B)** EP repetitions. **(C)** Perseverations. **(D)** AP times. Data are expressed as mean ± SEM. The asterisks indicate the significance level of *post hoc* comparisons among groups (*P < 0.05; ** *P* < 0.01; *** *P* < 0.005). DP: detection phase; EP: exercise phase; AP: automatization phase.

In TE1, unlike WS participants, PWS participants did not differ from TD children in DP errors they performed in detecting the sequence by doing (Figure 2A). Conversely, in comparison with TD and WS participants, PWS participants performed a number of DP errors significantly higher in OBS1 but not significantly different in OBS2 and TE2 tasks (Figure 2A), as revealed by *post hoc* comparisons on the second-order interaction of the three-way ANOVA (group × condition × task) (*F*_(2, 70)_ = 5.13, *P* = 0.0083, *η*_*P*_^2^ = 0.13).

As for EP repetitions, while WS participants needed a significantly higher number in comparison to TD participants, PWS and TD participants did not differ as revealed by *post hoc* comparisons made on the group effect (*F*_(2, 70)_ = 3.36, *P* = 0.040, *η*_*P*_^2^ = 0.09) of the three-way ANOVA (group × condition × task) (Figure 2B). Even the analysis of perseverations revealed no significant difference among PWS and TD participants. Conversely, in TE1, WS individuals performed a number of perseverations significantly higher than PWS and TD participants, as revealed by *post hoc* comparisons on the second-order interaction (*F*_(2, 70)_ = 3.18, *P* = 0.048, *η*_*P*_^2^ = 0.08) of the three-way ANOVA (group × condition × task) (Figure 2C).

A similar pattern was found in the analysis of the three AP times. PWS participants exhibited AP times significantly lower than WS individuals, but not significantly different from those of TD children, as revealed by *post hoc* comparisons on the group effect (*F*_(2, 70)_ = 8.26, *P* = 0.0006, *η*_*P*_^2^ = 0.19) of the four-way ANOVA (group × condition × task × time) (Figure 2D). All participants exhibited significantly reduced times as the task proceeded (*F*_(2, 140)_ = 33.67, *P* < 0.000001, *η*_*P*_^2^ = 0.32), indicating a progressive automatization of the task.

### Analysis of error

**Figure 3:**
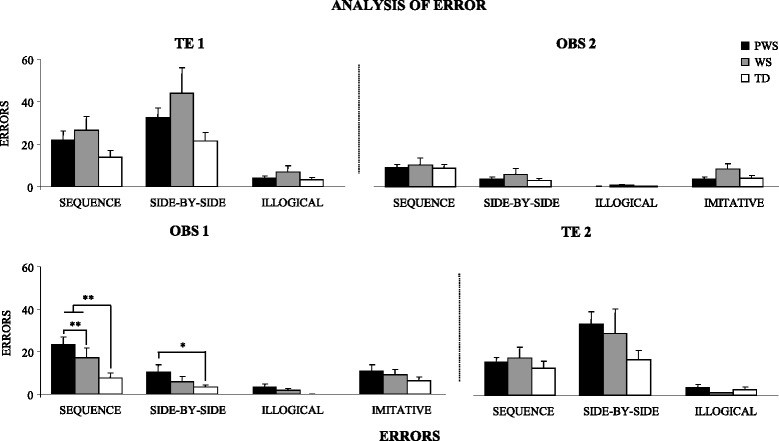
**Errors exhibited by PWS, WS, and TD participants in the two experimental conditions.** Data are expressed as mean ± SEM. The asterisks indicate the significance level of *post hoc* comparisons among groups (*P < 0.05; **P < 0.01).

**Figure 4:**
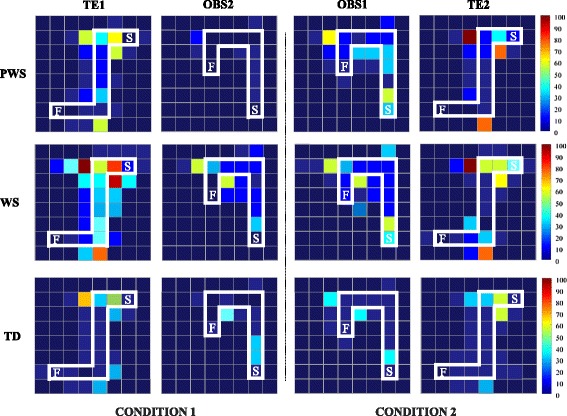
**Incorrect items touched on the screen by PWS, WS, and TD participants in performing the tasks.** On the right, the chromatic scale indicates the sum of incorrectly touched items (brown and blue denote maximal and minimal values, respectively). F: final point; S: starting point.

In OBS1, PWS individuals exhibited a number of sequence errors higher than TD children and interestingly higher than WS participants, as revealed by *post hoc* comparisons made on the significant interaction (*F*_(6, 105)_ = 2.93, *P* = 0.011, *η*_*P*_^2^ = 0.14) of the two-way ANOVA (group × type of error). The PWS individuals exhibited also a number of side-by-side errors higher than TD children. PWS, WS, and TD participants did not differ in the number of illogical and imitative errors (Figures 3 and 4). The analysis of error in the remaining TE1, OBS2, and TE2 tasks revealed no significant difference among the groups, even if significant differences among errors were found (always *P* < 0.000001) (Figures 3 and 4). Also interactions were not significant.

### Cognitive mapping abilities

No significant difference among groups and among error categories was found in any sequence (always *P* > 0.3), a clear index of similar cognitive mapping abilities in all groups.

## Discussion

The current study aimed at analyzing learning by observation and learning by doing in PWS in comparison with WS and TD individuals. With the exception of the imitative competencies, both visuo-motor learning tasks required attentive and mnesic functions, sequencing abilities, planning, response inhibition, cognitive flexibility, good knowledge and anticipatory expectation of effects related to actions, goal-directed actions, and motor imagery allowing recombination of novel actions with novel effects [29-31,41]. The main result of the present study showed a specific PWS deficit in learning by observation. The observational training did not help PWS individuals to detect and encode information, such as rules of the task, correct moves, and goals they had to reach. PWS individuals were impaired in reproducing the previously observed visuo-motor sequence when the observational task was proposed at first (OBS1), while they were as efficient as the TD children in detecting a sequence by trial and error (TE1 and TE2) and in reproducing the previously observed sequence when proposed as a second task (OBS2). The learning pattern of PWS was the reverse of that of WS individuals who were severely impaired in detecting the visuo-motor sequence in TE1 and as efficient as TD children in OBS1.

The deficit in learning by observation found in PWS individuals may be related to the impairment in social functioning described in this population [17]. Studies on the social deficits present in PWS have focused on either high frequencies of stubbornness, disobedience, and obsessive/compulsive and ritualistic behaviors or solitary behaviors, social withdrawal, and poor peer relations [42-44]. Even the frequent temper outbursts of PWS individuals are attributed to their impaired capacity to understand the motivations of others in the social milieu [17]. The poor performance of PWS individuals in OBS1 suggests a specific incapacity to use the information provided by the actor. PWS individuals display difficulty in recognizing and interpreting social cues and situations on the Social Attribution Task that measures the specific abilities necessary for interpreting social information [17]. It has been suggested that in PWS individuals the few attributions of feeling in social relationships indicate their deficit in empathy and ‘theory of mind.’ Such a deficit may determine impairment in interpersonal processes that are crucial for developing social abilities and understanding another’s thoughts and perspectives [12,45]. In a functional neuroimaging study, the typical difficulties in interacting with peers and understanding social environment displayed by PWS individuals are related to the perfusion abnormalities of the anterior cingulum and the cingulate gyrus found in these individuals [46]. Conclusively, the impaired PWS performance in OBS1 can be at least partially attributed to their difficulties in processing social information. The specular learning pattern of WS individuals (impaired TE1 and efficient OBS1) was coherent with their spared social abilities [47]. The PWS good performances in OBS2 do not contradict such an interpretation, because any second task (OBS2 and TE2) allowed overcoming the specific deficits of the clinical populations, by taking advantage of the previous experience (practice effect).

Once detected, the visuo-motor sequence had to be repeated until the error-free performance (exercise phase). The exercise phase mainly required working memory, memory load to form and maintain the trace of the correct sequence, long-term memory, and attentional demands to monitor its correct execution. Therefore, the efficient EP performance of PWS but not WS participants indicates a sparing of these abilities. Such a result complements the indication that the visuo-spatial domain is a strength point of PWS individuals [9,48,49]. Actually, the already-described PWS competence in solving spatial tasks, as for example jigsaw puzzles [50], may represent an advantage in performing the exercise phases. Also, the competent cognitive mapping abilities we found in PWS individuals point to this direction. The specular findings obtained by PWS and WS participants in EPs are related to the respective cognitive profiles. Indeed, the visuo-spatial domain is a strength point in PWS and conversely a strong weakness in WS. The WS deficits in spatial working and long-term memory [10,51-55] heavily impaired performances in all EPs. Finally, the PWS performances harmonize with the good capacity of spatial learning and localizatory memory shown by an animal model deficient of Necdin, a candidate gene in PWS etiology [56].

As for the kind of errors, all participants made an analogously low number of illogical errors, indicating that they all similarly managed the task fundamentals. Despite the specific deficit in observational learning of PWS participants, no difference in imitative errors was found among the groups. This result indicates that the imitative PWS deficit was not accompanied by a tendency to hyperimitate. The hyperimitation may be considered a tendency to affiliate or establish, maintain, and enhance relationships with the other. It may be linked to an ingratiating behavior that enhances the conformity with others [57]. Consistently, more empathic individuals and people scoring high in measures of social motivation tend to imitate [58,59]. Interestingly, PWS individuals are often hostile, with social withdrawal, put less emphasis on managing their social image, and exhibit scarce social motivation. Thus, the reduced number of imitative errors performed by PWS individuals is consistent with the social interpretation of their deficits in learning by observation. Given that people learn a lot through social interactions, the role of social motivation in the observational learning and whether a reduced social motivation may lead to impaired learning are interesting issues requiring future studies aimed to address which ways may boost learning.

In OBS1, PWS participants in comparison with TD children made more sequence and side-by-side errors when a change of direction was required. Errors in stopping the easier ‘keep-straight’ response and performing the more demanding ‘turn-left’ response resulted in the PWS participants’ difficulty suppressing a previously correct but then inappropriate response. Not by chance, correctly responding requires executive control processes based on frontal function, as response inhibition, cognitive flexibility, and attentional shifting [60,61], which are already indicated to be impaired in PWS [11,62-65]. Only a few studies have investigated brain abnormalities in PWS individuals; however, it is suggested that their executive dysfunction may be associated with fronto-parietal abnormalities [65-67]. The current findings can be nicely related to those obtained in an animal PWS model with a defect in the imprinting center, in which impaired abilities related to frontal abnormalities have been described in a five-choice serial reaction time task [68,69].

In the automatization phases, while WS participants displayed slowed down automatization times, PWS and TD participants showed similar times that progressively declined as the task was repeated. Specifically, the automatization phase required automatization of sequential visuo-motor productions to increase the efficiency and speed of the response and to achieve the highest levels of performance [70]. Automatizing skills are mainly linked to the functions of subcortical structures, such as the cerebellum and basal ganglia, and to their bidirectional interconnections with cortical structures [71-73]. Therefore, the efficient automatization in PWS indicates the preserved functionality of these brain networks. Similarly, the impaired WS automatization is consistent with brain abnormalities characterized by remarkable hypoplasia of the basal ganglia and the disproportionate enlargement of the cerebellum [74-77].

The performances of PWS individuals improved dramatically in OBS2, indicating the beneficial practice effect on the ability to learn by observation. Notably, the production of actions has a strong impact on action memory, so producing actions helps remember them [78]. Thus, actively produced actions influence the accessibility of memories by enhancing both the content and strength of the memory representation [79]. In this study, others’ actions appear to be linked to self-performed actions, as if agentive experience were functioning as a catalyst for action observation [80,81].

It should be emphasized that PWS individuals have language difficulties [16] that could impair their comprehension of verbal task instructions. However, the efficient performances of PWS individuals in the TE1 task (explained by means of exactly the same verbal instructions) indicated that their poor performances in OBS1 were not caused by a failure to understand the verbal instructions. If that were the case, both first tasks (OBS1 and TE1) would have been compromised.

Finally, a critical point in interpreting our results rests on our choice to adopt a between-group design, which meant that the conclusions were based on the performance of two different groups of PWS individuals. Although this design has some limitations in respect to the within-group design, we retained that a between-group design was adapted to address the differences between learning modalities. In fact, submitting people to various visuo-motor learning tasks inevitably implies a practice effect (learning effect), being difficult to propose them on different occasions, to render them different enough not to expect a change resulting from repeated testing, and to present them fully counterbalanced.

The present results could have important implications for developing interventions aimed at improving learning. In school, teaching is generally based on first showing how a task should be executed and then allowing for actual performance. The present data indicate that a useful way to improve learning in PWS individuals could be to use the ‘trick’ of first allowing them to actually perform a task and then eventually showing them how to refine the task that they have just experienced.

## Conclusions

The present study compared two learning mechanisms, learning by observation involving social processing and learning by doing involving direct experience, with all the other variables (for example, motor and cognitive complexity) being equal. A specific PWS deficit in learning by observation was found. Specifically, in comparison to WS and TD groups, PWS individuals were impaired in reproducing the previously observed visuo-motor sequence when the observational task was proposed first (OBS1), while they were as efficient as the TD children in detecting a sequence by trial and error (TE1 and TE2) and in reproducing the previously observed sequence when proposed as a second task (OBS2). We propose that the observational learning deficit in PWS individuals may be rooted, at least partially, in their incapacity to understand and/or use social information. As emphasized by Dimitropoulos et al. [16], there is increasing acknowledgement of social difficulties of PWS individuals above and beyond what is thought to be experienced by a person with a similar level of intellectual impairment. The characterization of their behavioral and cognitive phenotype allows for more targeted interventions aimed at stimulating and improving learning performances.

## Additional files

Additional file 1: Table S1. Statistical comparisons of performances of new WS and old WS. To verify the stability of WS participants’ data between the sample employed in the previously published study [30] and the present one, we compared the performances displayed by the 16 WS of the present study (new WS) and those by the 20 WS of the previously published study [30] (old WS) on the four main parameters by means of ANOVAs. Additional file 2: Table S2. Description of the tests used in the neuropsychological assessment. a. Statistical comparisons (one-way ANOVA) of performances between PWS (PWS1 and PWS2), WS (WS1 and WS2), and TD (TD1 and TD2) subgroups that performed the two experimental conditions. b. Statistical comparisons (one-way ANOVA) of performances of PWS1, WS1, and TD1 participants (Condition 1). c. Statistical comparisons (one-way ANOVA) of performances of PWS2, WS2, and TD2 participants (Condition 2). Additional file 3:
**Cognitive mapping abilities.** The protocol used to analyze the cognitive mapping abilities. At the end of each sequence, participants were required to draw the arrangement of the sequence that they had performed. The black square indicated the starting point of the OBS and TE tasks, respectively. Additional file 4: Table S4. The complete statistical analyses of performances of PWS, WS, and TD participants (three-way ANOVA: group × condition × task). In this analysis and in the subsequent ones, the differences statistically significant are indicated in bold. Additional file 5: Table S5. Analysis of covariance (ANCOVA). The complete statistical analyses of performances of PWS, WS, and TD participants (three-way ANCOVA: group × condition × task; covariates: mental age, chronological age, and IQ). In this analysis and in the subsequent ones, the differences statistically significant are indicated in bold.

## Abbreviations

AP: automatization phase
CA: chronological age
DP: detection phase
EP: exercise phase
ID: intellectual disability
MA: mental age
OBS: observational task
PWS: Prader-Willi syndrome
TD: typically developing
TE: trial and error task
WS: Williams syndrome
