# Supplementary material for: Learning by observation and learning by doing in Prader-Willi syndrome
Source: J Neurodev Disord. 2015 Feb 26;7(1):6. doi: 10.1186/s11689-015-9102-0 (PMC4409733; doi:10.1186/s11689-015-9102-0)
Supplement: Additional file 1: Table S1. — Statistical comparisons of performances of new WS and old WS. To verify the stability of WS participants’ data between the sample employed in the previously published study [30] and the present one, we compared the performances displayed by the 16 WS of the present study (new WS) and those by the 20 WS of the previously published study [30] (old WS) on the four main parameters by means of ANOVAs. [file 11689_2015_9102_MOESM1_ESM.doc]

**Table S1**

**Statistical comparisons of performances of New WS and Old WS**

To verify the stability of WS participants data between the sample employed in the previously published study (Foti *et al.* 2013) and the present one, we compared the performances displayed by the sixteen WS of the present study (New WS) and those of the twenty WS (Old WS) of the previously study (Foti *et al.* 2013) on the four main parameters by means of ANOVAs.

| **Parameters** | **Group effect**  ***F*_(1, 32)_**  ***p***  ***η_P_^2^*** | **Condition effect**  ***F*_(1, 32)_**  ***p***  ***η_P_^2^*** | **Task effect**  ***F*_(1, 32)_**  ***p***  ***η_P_^2^*** | **Interaction**  **(group x condition)**  ***F*_(1, 32)_**  ***p***  ***η_P_^2^*** | **Interaction**  **(group x task)**  ***F*_(1, 32)_**  ***p***  ***η_P_^2^*** | **Interaction**  **(condition x task)**  ***F*_(1, 32)_**  ***p***  ***η_P_^2^*** | **Interaction**  **(group x condition x task)**  ***F*_(1, 32)_**  ***p***  ***η_P_^2^*** |
| --- | --- | --- | --- | --- | --- | --- | --- |
| **DP errors** | *F* = 0.24  *p =* 0.62  *η_P_^2^* = 0.008 | *F* = 11.34  *p =* 0.002  *η_P_^2^* = 0.26 | *F* = 34.04  *p =* 0.0001  *η_P_^2^* = 0.52 | *F* = 0.0000002  *p =* 0.99  *η_P_^2^* = 0. 0000001 | *F* = 0.16  *p =* 0.69  *η_P_^2^* = 0.005 | *F* = 35.99  *p =* 0.000001  *η_P_^2^* = 0.53 | *F* = 0.001  *p =* 0.97  *η_P_^2^* = 0.00004 |
| **EP repetitions** | *F* = 0.19  *p =* 0.66  *η_P_^2^* = 0.006 | *F* = 0.75  *p =* 0.39  *η_P_^2^* = 0.02 | *F* = 1.58  *p =* 0.21  *η_P_^2^* = 0.05 | *F* = 0.06  *p =* 0.81  *η_P_^2^* = 0.002 | *F* = 0.009  *p =* 0.92  *η_P_^2^* = 0.0003 | *F* = 0.22  *p =* 0.64  *η_P_^2^* = 0.007 | *F* = 0.0007  *p =* 0.97  *η_P_^2^* = 0.00002 |
| **Perseverations** | *F* = 0.36  *p =* 0.55  *η_P_^2^* = 0.01 | *F* = 2.30  *p =* 0.13  *η_P_^2^* = 0.07 | *F* = 3.42  *p =* 0.07  *η_P_^2^* = 0.09 | *F* = 0.005  *p =* 0.94  *η_P_^2^* = 0.0001 | *F* = 0.007  *p =* 0.93  *η_P_^2^* = 0.0002 | *F* = 19.53  *p =* 0.0001  *η_P_^2^* = 0.38 | *F* = 0.22  *p =* 0.63  *η_P_^2^* = 0.007 |
| **AP time 3** | *F* = 0.03  *p =* 0.86  *η_P_^2^* = 0.001 | *F* = 0.15  *p =* 0.69  *η_P_^2^* = 005 | *F* = 0.25  *p =* 0.61  *η_P_^2^* = 0.008 | *F* = 0.04  *p =* 0.84  *η_P_^2^* = 0.001 | *F* = 0.01  *p =* 0.90  *η_P_^2^* = 0.0004 | *F* = 10.79  *p =* 0.002  *η_P_^2^* = 0.25 | *F* = 0.06  *p =* 0.79  *η_P_^2^* = 0.002 |

**Performances of New WS, Old WS, and Shared WS**

Mean performances of New WS (16 WS participants of the present research), Old WS (20 WS participants of the research by Foti *et al.* 2013), and Shared WS (8 WS participants of the both researches).

|  | **DP errors**  Mean  (± SEM)  **OBS TE** | | **EP repetitions**  Mean  (± SEM)  **OBS TE** | | **Perseverations**  Mean  (± SEM)  **OBS TE** | | **AP time 3**  Mean  (± SEM)  **OBS TE** | |
| --- | --- | --- | --- | --- | --- | --- | --- | --- |
| **New WS (8)**  (Condition 1) | 8.5  (± 1.6) | 54.3  (± 10.3) | 9.1  (± 2.7) | 11.9  (± 3.7) | 4.7  (± 1.7) | 14.5  (± 4.7) | 11062.7  (± 2081.7) | 13108.4  (± 1909.9) |
| **New WS (8)**  (Condition 2) | 16.5  (± 3.4) | 14.4  (± 2.9) | 7.1  (± 2.4) | 8.4  (± 3.2) | 7.1  (± 2.2) | 3.4  (± 1.2) | 12951.2  (± 3430.7) | 10406.2  (± 1608.8) |
| **Old WS (10)**  (Condition 1) | 9.1  (± 1.8) | 58.5  (± 11.6) | 9.4  (± 2.7) | 12.6  (± 3.1) | 5.7  (± 2.1) | 17.5  (± 6.1) | 11351.6  (± 1959.4) | 12908.5  (± 1741.9) |
| **Old WS (10)**  (Condition 2) | 17.4  (± 3.5) | 18.2  (± 3.8) | 8.7  (± 3.0) | 10.2  (± 3.1) | 9.4  (± 3.5) | 4.3  (± 1.6) | 12079.8  (± 2782.9) | 9709.3  (± 1351.8) |
| **Shared WS (4)**  (Condition 1) | 8.4  (± 1.7) | 50.2  (± 10.9) | 7.7  (± 2.4) | 10.9  (± 2.8) | 5.5  (± 1.8) | 14.2  (± 5.4) | 11357.9  (±1662.9) | 12204.3  (±1507.2) |
| **Shared WS (4)**  (Condition 2) | 16.35  (± 3.38) | 18.7  (± 3.8) | 8.4  (± 2.6) | 8.6  (± 2.6) | 8.4  (± 3.1) | 5.7  (± 1.7) | 11496.2  (±2331.7) | 9718.6  (±1141.4) |
