# Supplementary material for: Learning by observation and learning by doing in Prader-Willi syndrome
Source: J Neurodev Disord. 2015 Feb 26;7(1):6. doi: 10.1186/s11689-015-9102-0 (PMC4409733; doi:10.1186/s11689-015-9102-0)
Supplement: Additional file 2: Table S2. — Description of the tests used in the neuropsychological assessment. a. Statistical comparisons (one-way ANOVA) of performances between PWS (PWS1 and PWS2), WS (WS1 and WS2), and TD (TD1 and TD2) subgroups that performed the two experimental conditions. b. Statistical comparisons (one-way ANOVA) of performances of PWS1, WS1, and TD1 participants (Condition 1). c. Statistical comparisons (one-way ANOVA) of performances of PWS2, WS2, and TD2 participants (Condition 2). [file 11689_2015_9102_MOESM2_ESM.doc]

**Table S2**

Description of the tests used in the neuropsychological assessment.

| **Cognitive domain** | **Description** |
| --- | --- |
| Visuo-Motor Integration  (VMI) | The test consists of a sequence of 27 geometric shapes ranging from simple to difficult. The subject has to copy each shape on a sheet of paper using a pencil. The score is the number of correct shapes copied (maximum score: 27). |
| Visuo-Spatial Short-term memory  (VSS) | The material consists of a non-verbalizable geometric shape depicted in high contrast colors (green–red) that appears for two seconds in one of seven possible positions on the computer screen. After one second (ISI), the same geometric shape appears in a second position and then disappears after 2 seconds. After 500 msec (delay interval), two empty cells (2 × 3 cm) are presented in the same spatial positions as before and the subject has to indicate the order in which the stimuli appeared. If the subject is successful in at least 3 of 5, two-position sequences, a sequence one block longer is presented. If the subject fails (less than 3 correct answers in a block), the task is discontinued. The same testing procedure is used for sequences of increasing length (up to a maximum of 7 spatial positions). |
| Visuo-Object Short-term memory  (VOS) | The material consists of 7 complex geometric figures depicted in high contrast colors (green–red). At the onset of the task, 2 figures are presented, one at time, for 2 seconds at the center of the computer screen with a 1-sec ISI; 500 msec after the disappearance of the second figure, the two stimulus figures are presented aligned in the center of the screen in a random position (left *vs.* right) and the participant is asked to indicate the order in which they appeared. If the subject is successful in at least 3 of the 5 trials, a sequence one figure longer is presented and the task continues until a maximum of 7 figures have been presented. |

1. Statistical comparisons (one-way ANOVA) of performances between PWS (PWS1 and PWS2), WS (WS1 and WS2), and TD (TD1 and TD2) subgroups that performed the two experimental conditions.

| Group | VMI  Mean  (± SEM) | *F*_(fd)_  *P*  *η_P_^2^* | VSS  Mean  (± SEM) | *F*_(fd)_  *p*  *η_P_^2^* | VOS  Mean  (± SEM) | *F*_(fd)_  *p*  *η_P_^2^* |
| --- | --- | --- | --- | --- | --- | --- |
| PWS1  (Condition 1) | 13.00  (± 0.78) | *F*_1, 22_ = 0.02  *p* = 0.88  *η_P_^2^* = 0.001 | 3.40  (± 0.25) | *F*_1, 22_ = 0.09  *p* = 0.75  *η_P_^2^* = 0.004 | 2.67  (± 0.22) | *F*_1, 22_ = 0.71  *p* = 0.41  *η_P_^2^* = 0.031 |
| PWS2  (Condition 2) | 13.17  (± 0.79) |  | 3.30  (± 0.17) |  | 2.92  (± 0.19) |  |
| WS1  (Condition 1) | 12.83  (± 0.81) | *F*_1, 22_ = 0.006  *p* = 0.94  *η_P_^2^* = 0.0003 | 2.58  (± 0.29) | *F*_1, 22_ = 0.04  *p* = 0.84  *η_P_^2^* = 0.002 | 2.50  (± 0.15) | *F*_1, 22_ = 2.89  *p* = 0.10  *η_P_^2^* = 0.12 |
| WS2  (Condition 2) | 12.75  (± 0.71) |  | 2.67  (± 0.28) |  | 2.92  (± 0.19) |  |
| TD1  (Condition 1) | 15.29  (± 0.38) | *F*_1, 26_ = 0.25  *p* = 0.62  *η_P_^2^* = 0.01 | 3.64  (± 0.20) | *F*_1, 26_ = 1.71  *p* = 0.20  *η_P_^2^* = 0.06 | 2.93  (± 0.20) | *F*_1, 26_ = 0.073  *p* = 0.79  *η_P_^2^* = 0.0038 |
| TD2  (Condition 2) | 15.00  (± 0.42) |  | 3.21  (± 0.26) |  | 2.86  (± 0.18) |  |

VMI, Visuo-Motor Integration; VSS, Visuo-Spatial Short-term memory; VOS, Visuo-Object Short-term memory.

**b.** Statistical comparisons (one-way ANOVA) of performances of PWS1, WS1 and TD1 participants (Condition 1).

| Cognitive domain | PWS1  Mean  (± SEM) | WS1  Mean  (± SEM) | TD1  Mean  (± SEM) | Group effect  *F*_(fd)_  *p*  *η_P_^2^* | Post hoc  Newman-Keuls test  *p; Cohen’s d; r* |
| --- | --- | --- | --- | --- | --- |
| VMI | 13.00  (± 0.78) | 12.83  (± 0.81) | 15.29  (± 0.38) | *F*_2, 35_ = 4.53  *p* = 0.02  *η_P_^2^* = 0.21 | PWS vs. WS *p* = 0.85  *d* = 0.06; *r* = 0.03  PWS vs. TD *p* = 0.02  *d* = -1.06; *r* = -0.47  WS vs. TD *p* = 0.03  *d* = -1.11; *r* = -0.48 |
| VSS | 3.40  (± 0.25) | 2.58  (± 0.29) | 3.64  (± 0.20) | *F*_2, 35_ = 5.13  *p* = 0.01  *η_P_^2^* = 0.23 | PWS vs. WS *p* = 0.02  *d* = 0.87; *r* = 0.40  PWS vs. TD *p* = 0.49  *d* = -0.29; *r* = -0.15  WS vs. TD *p* = 0.01  *d* = -1.20; *r* = -0.52 |
| VOS | 2.67  (± 0.22) | 2.50  (± 0.15) | 2.93  (± 0.20) | *F*_2, 35_ = 1.29  *p* = 0.29  *η_P_^2^* = 0.07 |  |

VMI, Visuo-Motor Integration; VSS, Visuo-Spatial Short-term memory; VOS, Visuo-Object Short-term memory.

**c.** Statistical comparisons (one-way ANOVA) of performances of PWS2, WS2 and TD2 participants (Condition 2).

| Cognitive domain | PWS2  Mean  (± SEM) | WS2  Mean  (± SEM) | TD2  Mean  (± SEM) | Group effect  *F*_(fd)_  *p*  *η_P_^2^* | Post hoc  Newman-Keuls test  *p; Cohen’s d; r* |
| --- | --- | --- | --- | --- | --- |
| VMI | 13.17  (± 0.79) | 12.75  (± 0.71) | 15.00  (± 0.42) | *F*_2, 35_ = 3.69  *p* = 0.03  *η_P_^2^* = 0.17 | PWS vs. WS *p* = 0.64  *d* = 0.16; *r* = 0.08  PWS vs. TD *p* = 0.04  *d* = -0.82; *r* = -0.38  WS vs. TD *p* = 0.04  *d* = -1.09; *r* = -0.48 |
| VSS | 3.30  (± 0.17) | 2.67  (± 0.28) | 3.21  (± 0.26) | *F*_2, 35_ = 5.01  *p* = 0.01  *η_P_^2^* = 0.22 | PWS vs. WS *p* = 0.04  *d* = 0.77; *r* = 0.36  PWS vs. TD *p* = 0.29  *d* = 0.11; *r* = 0.05  WS vs. TD *p* = 0.01  *d* = -0.55; *r* = -0.27 |
| VOS | 2.92  (± 0.19) | 2.93  (± 0.19) | 2.86  (± 0.18) | *F*_2, 35_ = 0.03  *p* = 0.96  *η_P_^2^* = 0.002 |  |

VMI, Visuo-Motor Integration; VSS, Visuo-Spatial Short-term memory; VOS, Visuo-Object Short-term memory.
