# Supplementary material for: Learning by observation and learning by doing in Prader-Willi syndrome
Source: J Neurodev Disord. 2015 Feb 26;7(1):6. doi: 10.1186/s11689-015-9102-0 (PMC4409733; doi:10.1186/s11689-015-9102-0)
Supplement: Additional file 3: — Cognitive mapping abilities. The protocol used to analyze the cognitive mapping abilities. At the end of each sequence, participants were required to draw the arrangement of the sequence that they had performed. The black square indicated the starting point of the OBS and TE tasks, respectively. [file 11689_2015_9102_MOESM3_ESM.doc]

**Cognitive mapping abilities**

Protocol used to analyze the cognitive mapping abilities. At the end of each sequence, participants were required to draw the arrangement of the sequence that they had performed. The black square indicated the starting point of the OBS and TE task, respectively.

OBS task

|  |  |  |  |  |  |  |  |
| --- | --- | --- | --- | --- | --- | --- | --- |
|  |  |  |  |  |  |  |  |
|  |  |  |  |  |  |  |  |
|  |  |  |  |  |  |  |  |
|  |  |  |  |  |  |  |  |
|  |  |  |  |  |  |  |  |
|  |  |  |  |  |  |  |  |
|  |  |  |  |  |  |  |  |

TE task

|  |  |  |  |  |  |  |  |
| --- | --- | --- | --- | --- | --- | --- | --- |
|  |  |  |  |  |  |  |  |
|  |  |  |  |  |  |  |  |
|  |  |  |  |  |  |  |  |
|  |  |  |  |  |  |  |  |
|  |  |  |  |  |  |  |  |
|  |  |  |  |  |  |  |  |
|  |  |  |  |  |  |  |  |
