# Supplementary material for: Learning by observation and learning by doing in Prader-Willi syndrome
Source: J Neurodev Disord. 2015 Feb 26;7(1):6. doi: 10.1186/s11689-015-9102-0 (PMC4409733; doi:10.1186/s11689-015-9102-0)
Supplement: Additional file 5: Table S5. — Analysis of covariance (ANCOVA). The complete statistical analyses of performances of PWS, WS, and TD participants (three-way ANCOVA: group × condition × task; covariates: mental age, chronological age, and IQ). In this analysis and in the subsequent ones, the differences statistically significant are indicated in bold. [file 11689_2015_9102_MOESM5_ESM.doc]

**Table S5 - Analysis of covariance (ANCOVA)**

**The complete statistical analyses of performances of PWS, WS and TD participants (three-way ANCOVA: group x condition x task; covariates: mental age, chronological age and QI). In this analysis and in the subsequent ones, the differences statistically significant are indicated in bold.**

| **Parameters** | **Group effect**  ***F*_(2, 67)_**  ***p***  ***η_P_^2^*** | **Condition effect**  ***F*_(1, 67)_**  ***p***  ***η_P_^2^*** | **Task effect**  ***F*_(1, 70)_**  ***p***  ***η_P_^2^*** | **Interaction**  **(group x condition)**  ***F*_(2, 67)_**  ***p***  ***η_P_^2^*** | **Interaction**  **(group x task)**  ***F*_(2, 70)_**  ***p***  ***η_P_^2^*** | **Interaction**  **(condition x task)**  ***F*_(1, 70)_**  ***p***  ***η_P_^2^*** | **Interaction**  **(group x condition x task)**  ***F*_(2, 70)_**  ***p***  ***η_P_^2^*** |
| --- | --- | --- | --- | --- | --- | --- | --- |
| **DP errors** | *F* = 2.80  *p =* 0.06  *η_P_^2^* = 0.08 | *F* = 1.92  *p =* 0.17  *η_P_^2^* = 0.03 | ***F* = 52.52**  ***p <* 0.000001**  ***η_P_^2^* = 0.43** | *F* = 2.27  *p =* 0.11  *η_P_^2^* = 0.06 | *F* = 0.95  *p =* 0.39  *η_P_^2^* = 0.03 | ***F* = 29.14**  ***p <* 0.000001**  ***η_P_^2^* = 0.29** | ***F* = 5.14**  ***p =* 0.008**  ***η_P_^2^* = 0.13** |
| **EP repetitions** | ***F* = 3.49**  ***p =* 0.03**  ***η_P_^2^* = 0.09** | *F* = 0.76  *p =* 0.38  *η_P_^2^* = 0.01 | ***F* = 9.48**  ***p =* 0.003**  ***η_P_^2^* = 0.12** | *F* = 0.69  *p =* 0.50  *η_P_^2^* = 0.02 | *F* = 1.75  *p =* 0.18  *η_P_^2^* = 0.05 | *F* = 0.33  *p =* 0.57  *η_P_^2^* = 0.005 | *F* = 2.48  *p =* 0.09  *η_P_^2^* = 0.07 |
| **Perseverations** | ***F* = 3.11**  ***p =* 0.05**  ***η_P_^2^* = 0.08** | *F* = 0.22  *p =* 0.64  *η_P_^2^* = 0.003 | ***F* = 5.34**  ***p =* 0.02**  ***η_P_^2^* = 0.07** | *F* = 0.58  *p =* 0.56  *η_P_^2^* = 0.02 | *F* = 0.18  *p =* 0.83  *η_P_^2^* = 0.005 | ***F* = 7.72**  ***p =* 0.007**  ***η_P_^2^* = 0.09** | ***F* = 3.04**  ***p =* 0.04**  ***η_P_^2^* = 0.08** |

**Below the post-hoc comparisons following the higher significant effect of the three-way ANCOVA (group x condition x task; covariates: mental age, chronological age and QI) for each parameter were reported.**

*Post-hoc comparisons on the Group effect*

| **EP repetitions**  *p; Cohen’s d; r* | |
| --- | --- |
| **PWS *vs.* TD** | *p =* 0.19; *d* = 0.33; *r* = 0.16 |
| **PWS *vs.* WS** | *p =* 0.21; *d* = -0.26; *r* = -0.13 |
| **WS *vs.* TD** | ***p =* 0.03;** *d* = -0.57; *r* = -0.27 |

*Post-hoc comparison* on the Task effect

| **EP repetitions**  *p; Cohen’s d; r* | |
| --- | --- |
| **OBS 1 and 2 *vs.* TE 1 and 2** | ***p =* 0.003;** *d* = -0.38; *r* = -0.19 |

*Post-hoc comparisons on the second-order interaction of the three-way ANC*OVA (group x condition x task)

| **Groups** | **DP errors** | | | |
| --- | --- | --- | --- | --- |
|  | **Condition 1** | | **Condition 2** | |
|  | **TE1**  *p; Cohen’s d; r* | **OBS2**  *p; Cohen’s d;* *r* | **OBS1**  *p*; *Cohen’s d; r* | **TE2**  *p*; *Cohen’s d; r* |
| **PWS *vs.* TD** | *p =* 0.06  *d* = 0.74; *r* = 0.35 | *p =* 0.70  *d* = 0.29; *r* = 0.14 | ***p =* 0.002**  *d* = 2.11; *r* = 0.72 | *p =* 0.25  *d* = 0.36; *r* = 0.18 |
| **PWS *vs.* WS** | *p =* 0.34  *d* = -0.19; *r* = -0.09 | *p =* 0.83  *d* = 0.80; *r* = 0.37 | ***p =* 0.04**  *d* = 1.32; *r* = 0.55 | *p =* 0.12  *d* = 1.03; *r* = 0.46 |
| **WS *vs.* TD** | ***p =* 0.009**  *d* = -0.64; *r* = -0.30 | *p =* 0.88  *d* = 0.38; *r* = 0.18 | *p =* 0.62  *d* = -0.73; *r* = -0.34 | *p =* 0.41  *d* = 0.26; *r* = 0.13 |

| **Groups** | **Perseverations** | | | |
| --- | --- | --- | --- | --- |
|  | **Condition 1** | | **Condition 2** | |
|  | **TE1**  *p; Cohen’s d; r* | **OBS2**  *p; Cohen’s d;* *r* | **OBS1**  *p*; *Cohen’s d;* *r* | **TE2**  *p*; *Cohen’s d;* *r* |
| **PWS *vs.* TD** | *p =* 0.93  *d* = 0.41; *r* = 0.19 | *p =* 0.64  *d* = -0.38; *r* = -0.19 | *p =* 0.94  *d* = 0.32; *r* = 0.16 | *p =* 0.98  *d* = -0.06; *r* = -0.03 |
| **PWS *vs.* WS** | ***p =* 0.006**  *d* = -0.55; *r* = -0.26 | *p =* 0.76  *d* = -0.82; *r* = -0.38 | *p =* 0.51  *d* = -0.47; *r* = -0.23 | *p =* 0.98  *d* = -0.28; *r* = -0.14 |
| **WS *vs.* TD** | ***p =* 0.002**  *d* = -0.74; *r* = -0.35 | *p =* 0.92  *d* = -0.52; *r* = -0.25 | *p =* 0.34  *d* = -0.65; *r* = -0.31 | *p =* 0.96  *d* = -0.16; *r* = -0.08 |

**AP times of PWS, WS and TD participants (four-way ANCOVA: group x condition x task x time; covariates: mental age, chronological age and QI**)

| **Parameters** | **Group effect (1)**  ***F*_(2, 67)_**  ***p***  ***η_P_^2^*** | **Condition effect (2)**  ***F*_(1, 67)_**  ***p***  ***η_P_^2^*** | **Task effect (3)**  ***F*_(1, 70)_**  ***p***  ***η_P_^2^*** | **Time effect(4)**  ***F*_(2, 140)_**  ***p***  ***η_P_^2^*** | **Interaction**  **(1 x 2)**  ***F*_(2, 67)_**  ***p***  ***η_P_^2^*** | **Interaction**  **(1 x 3)**  ***F*_(2, 70)_**  ***p***  ***η_P_^2^*** | **Interaction**  **(2 x 3)**  ***F*_(1, 70)_**  ***p***  ***η_P_^2^*** | **Interaction**  **(1 x 4)**  ***F*_(4, 140)_**  ***p***  ***η_P_^2^*** | **Interaction**  **(2 x 4)**  ***F*_(2, 140)_**  ***p***  ***η_P_^2^*** | **Interaction**  **(3 x 4)**  ***F*_(2, 140)_**  ***p***  ***η_P_^2^*** | **Interaction**  **(1 x 2 x 3)**  ***F*_(2, 70)_**  ***p***  ***η_P_^2^*** | **Interaction**  **(1 x 2 x 4)**  ***F*_(4, 140)_**  ***p***  ***η_P_^2^*** | **Interaction**  **(1 x 3 x 4)**  ***F*_(4, 140)_**  ***p***  ***η_P_^2^*** | **Interaction**  **(2 x 3 x 4)**  ***F*_(2, 140)_**  ***p***  ***η_P_^2^*** | **Interaction**  **(1 x 2 x 3 x 4)**  ***F*_(4, 140)_**  ***p***  ***η_P_^2^*** |
| --- | --- | --- | --- | --- | --- | --- | --- | --- | --- | --- | --- | --- | --- | --- | --- |
| **AP times** | ***F* = 8.37**  ***p =* 0.0006**  ***η_P_^2^* = 0.19** | *F* = 0.09  *p =* 0.75  *η_P_^2^* = 0.001 | ***F* = 4.17**  ***p =* 0.04**  ***η_P_^2^* = 0.06** | ***F* = 33.67**  ***p <* 0.000001**  ***η_P_^2^* = 0.32** | *F* = 0.54  *p =* 0.59  *η_P_^2^* = 0.015 | *F* = 0.14  *p =* 0.87  *η_P_^2^* = 0.004 | ***F* = 9.60**  ***p =* 0.003**  ***η_P_^2^* = 0.12** | ***F* = 2.99**  ***p =* 0.002**  ***η_P_^2^* = 0.08** | *F* = 0.63  *p =* 0.53  *η_P_^2^* = 0.009 | *F* = 1.11  *p =* 0.33  *η_P_^2^* = 0.01 | *F* = 0.32  *p =* 0.72  *η_P_^2^* = 0.009 | *F* = 0.25  *p =* 0.91  *η_P_^2^* = 0.007 | *F* = 1.46  *p =* 0.22  *η_P_^2^* = 0.04 | *F* = 1.09  *p =* 0.34  *η_P_^2^* = 0.01 | *F* = 0.23  *p =* 0.92  *η_P_^2^* = 0.006 |

Post-hoc comparisons on the Group effect

| **AP times**  *p*; *Cohen’s d; r* | |
| --- | --- |
| **PWS *vs.* TD** | *p =* 0.53; *d* = -0.17; *r* = -0.09 |
| **PWS *vs.* WS** | ***p =* 0.0008;** *d* = -0.68; *r* = -0.32 |
| **WS *vs.* TD** | ***p =* 0.002;** *d* = -0.55; *r* = -0.27 |

**Analysis of error [two-way ANCOVA: group (PWS, WS, TD) x type of error (illogical; sequence; side-by-side; imitative); covariates: mental age, chronological age and QI]**

| **Task** | **Group effect**  ***F*_(2, 32)_**  ***p***  ***η_P_^2^*** | **Type of error effect**  ***F*_(2, 70)_**  ***p***  ***η_P_^2^*** | **Interaction**  **(group x type of error)**  ***F*_(4, 70)_**  ***p***  ***η_P_^2^*** |
| --- | --- | --- | --- |
| **TE1** | *F* = 2.46  *p =* 0.64  *η_P_^2^* = 0.28 | ***F* = 42.05**  ***p <* 0.000001**  ***η_P_^2^* = 0.54** | *F* = 1.73  *p =* 0.15  *η_P_^2^* = 0. 09 |
| **OBS2** | *F* = 0.89  *p =* 0.42  *η_P_^2^* = 0.05 | ***F* = 28.03**  ***p <* 0.000001**  ***η_P_^2^* = 0.44** | *F* = 0.75  *p =* 0.61  *η_P_^2^* = 0.04 |
| **OBS1** | *F* = 1.32  *p =* 0.28  *η_P_^2^* = 0.08 | ***F* = 35.75**  ***p <* 0.000001**  ***η_P_^2^* = 0.50** | ***F* = 2.93**  ***p =* 0.01**  ***η_P_^2^* = 0.14** |
| **TE2** | *F* = 0.13  *p =* 0.88  *η_P_^2^* = 0.008 | ***F* = 28.12**  ***p <* 0.000001**  ***η_P_^2^* = 0.44** | *F* = 1.47  *p =* 0.22  *η_P_^2^* = 0.08 |

**Below the post-hoc comparisons following the higher significant effect of the two-way ANCOVA (group x type of error; covariates: mental age, chronological age and QI) for each task were reported.**

Post-hoc comparisons on the Type of error effect

| **TE1** | |
| --- | --- |
| Type of error | *p; Cohen’s d; r* |
| **illogical *vs.* sequence** | ***p =* 0.0001;** *d* = -0.19; *r* = -0.51 |
| **illogical *vs.* side-by-side** | ***p =* 0.0001;** *d* = -1.32; *r* = -0.55 |
| **sequence *vs.* side-by-side** | ***p =* 0.0004;** *d* = -0.49; *r* = -0.24 |

| **OBS2** | |
| --- | --- |
| Type of error | *p; Cohen’s d; r* |
| **illogical *vs.* sequence** | ***p =* 0.0001;** *d* = -1.57; *r* = -0.62 |
| **illogical *vs.* side-by-side** | ***p =* 0.0003;** *d* = -0.78; *r* = -0.36 |
| **illogical *vs.* imitative** | ***p =* 0.0001;** *d* = -1.01; *r* = -0.45 |
| **sequence *vs.* side-by-side** | ***p =* 0.0001;** *d* = 0.73; *r* = 0.34 |
| **sequence *vs.* imitative** | ***p =* 0.0002;** *d* = 0.57; *r* = 0.27 |
| **side-by-side *vs.* imitative** | *p =* 0.25; *d* = -0.18; *r* = -0.09 |

| **TE2** | |
| --- | --- |
| Type of error | *p; Cohen’s d; r* |
| **illogical *vs.* sequence** | ***p =* 0.0002;** *d* = -1.33; *r* = -0.55 |
| **illogical *vs.* side-by-side** | ***p =* 0.0001;** *d* = -1.21; *r* = -0.52 |
| **sequence *vs.* side-by-side** | ***p =* 0.0009;** *d* = -0.50; *r* = -0.24 |

*Post-hoc comparisons on the first-order interaction of the two-way ANCOVA (group x type of error; covariates: mental age, chronological age and QI*)

| **Groups** | **OBS1** | | | |
| --- | --- | --- | --- | --- |
|  | Type of error  *p; Cohen’s d; r* | | | |
|  | illogical | sequence | side-by-side | imitative |
| **PWS *vs.* TD** | *p =* 0.40  *d* = 0.87; *r* = 0.39 | ***p =* 0.0001**  *d* = 1.59; *r* = 0.62 | ***p =* 0.048**  *d* = 0.75; *r* = 0.35 | *p =* 0.27  *d* = 0.54; *r* = 0.26 |
| **PWS *vs.* WS** | *p =* 0.57  *d* = 0.34; *r* = 0.17 | ***p =* 0.009**  *d* = 0.44; *r* = 0.21 | *p =* 0.42  *d* = 0.39; *r* = 0.19 | *p =* 0.68  *d* = 0.21; *r* = 0.10 |
| **WS *vs.* TD** | *p =* 0.47  *d* = -0.94; *r* = -0.42 | ***p =* 0.002**  *d* = -0.70; *r* = -0.33 | *p =* 0.25  *d* = -0.42; *r* = -0.21 | *p =* 0.47  *d* = -0.35; *r* = -0.17 |
